# Large language models and synthetic health data: progress and prospects

**DOI:** 10.1093/jamiaopen/ooae114

**Published:** 2024-10-26

**Authors:** Daniel Smolyak, Margrét V Bjarnadóttir, Kenyon Crowley, Ritu Agarwal

**Affiliations:** Department of Computer Science, University of Maryland, College Park, College Park, MD 20742, United States; Robert H. Smith School of Business, University of Maryland, College Park, College Park, MD 20740, United States; Accenture Federal Services, Arlington, VA 22203, United States; Center for Digital Health and Artificial Intelligence, Carey Business School, Johns Hopkins University, Baltimore, MD 21202, United States

**Keywords:** large language models, generative artificial intelligence, synthetic data, health equity, responsible AI

## Abstract

**Objectives:**

Given substantial obstacles surrounding health data acquisition, high-quality synthetic health data are needed to meet a growing demand for the application of advanced analytics for clinical discovery, prediction, and operational excellence. We highlight how recent advances in large language models (LLMs) present new opportunities for progress, as well as new risks, in synthetic health data generation (SHDG).

**Materials and Methods:**

We synthesized systematic scoping reviews in the SHDG domain, recent LLM methods for SHDG, and papers investigating the capabilities and limits of LLMs.

**Results:**

We summarize the current landscape of generative machine learning models (eg, Generative Adversarial Networks) for SHDG, describe remaining challenges and limitations, and identify how recent LLM approaches can potentially help mitigate them.

**Discussion:**

Six research directions are outlined for further investigation of LLMs for SHDG: evaluation metrics, LLM adoption, data efficiency, generalization, health equity, and regulatory challenges.

**Conclusion:**

LLMs have already demonstrated both high potential and risks in the health domain, and it is important to study their advantages and disadvantages for SHDG.

## Introduction

The recent release of generative large language models (LLMs), such as OpenAI’s GPT models^1^ and Google’s PaLM^2^ has generated both robust enthusiasm as well as significant concern related to the use of generative artificial intelligence (AI) in healthcare.^3–5^ Numerous potential applications for healthcare have been documented, including processing of administrative data, such as discharge summary generation, interfacing as a chatbot with doctors for diagnosis or treatment determination, interfacing as a chatbot with patients for mental healthcare delivery, producing clinical trial documentation, intelligent tagging of patient images (eg, radiology or pathology images), and creation of educational health material.^6–14^ General-purpose LLMs have been found to achieve high performance on clinical licensing exams and comprehensive medical Q&A benchmarks,^15–17^ and LLMs trained on medical data have successfully augmented clinician diagnostic performance.^18^

**Figure 1.**
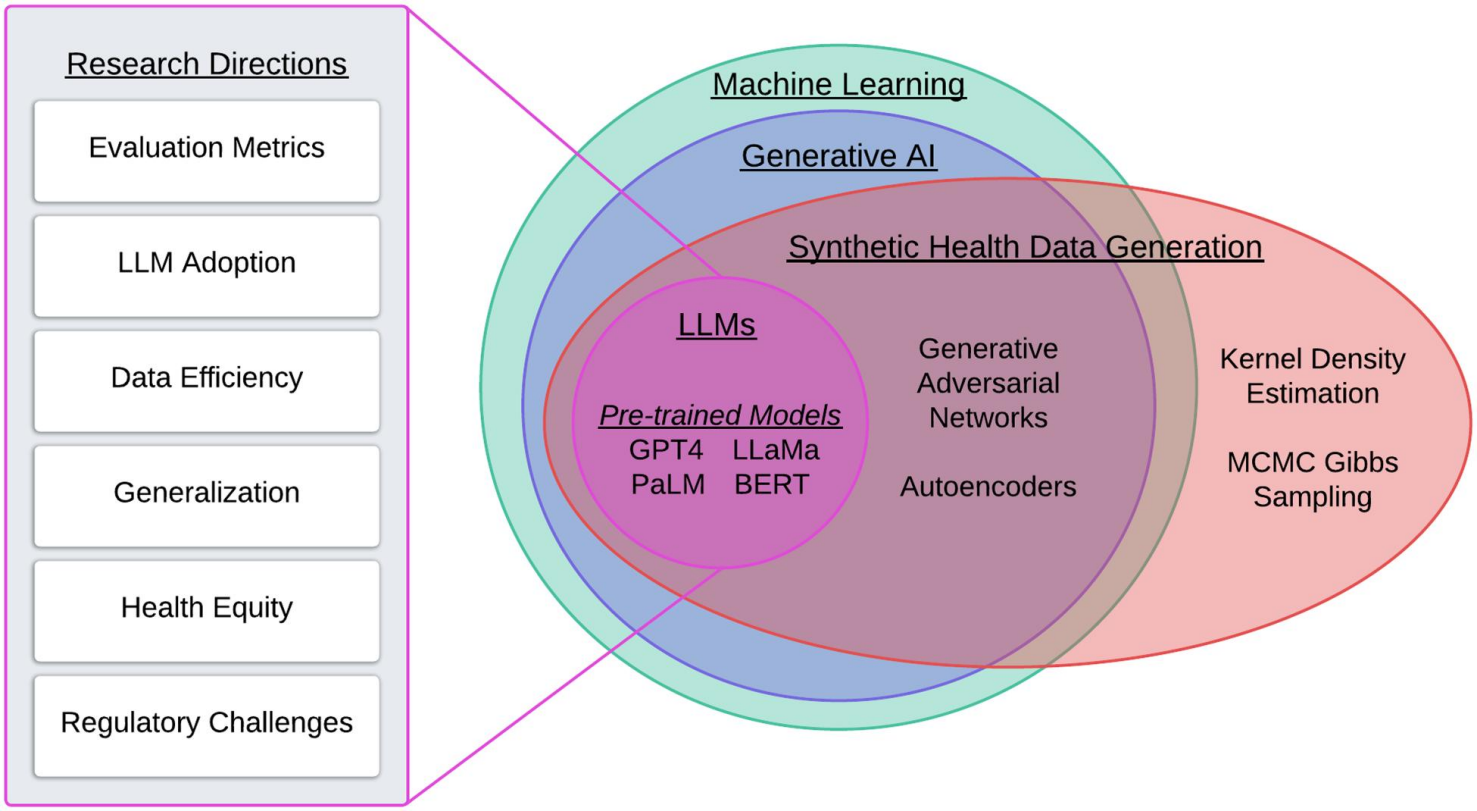
An overview of the concepts and research directions discussed in the article.

In this perspective, we focus on 1 particularly promising avenue for LLMs: the creation of synthetic health data. There is a significant need for augmented datasets, as health data are often limited in size, may be costly to collect, not representative of diverse populations, and privacy concerns limit its sharing.^19^^,^^20^ Machine learning models for prediction and classification often require large datasets, particularly for deep learning models common in health.^21^ Yet datasets can be insufficiently large due to the rapid evolution of diseases, such as COVID-19,^22^ rarity of disease,^23^ or the myriad obstacles to sharing and acquiring existing health data, including ethical, legal, political, economic, cultural, and technical barriers.^24^^,^^25^ Synthetic data provide a unique opportunity for health dataset expansion or creation, by addressing privacy concerns and other barriers. We build on existing research on the state of the art in synthetic health data generation (SHDG)^20^^,^^23^^,^^26–32^ and broad exploration of the potential risks and opportunities for LLMs in healthcare.^6^ We contribute to the literature with a focused assessment of LLMs for SHDG, including a review of early research in the area and recommendations for future research directions (see Figure 1 for a summary of the paper’s key concepts).

## Synthetic health data generation

Synthetic data can be characterized by a combination of their resemblance to and distance from real data—they aim to mimic the statistical distribution and usability of real data while restricting reidentification of original data points (ie, individuals). Common characteristics for assessing synthetic datasets include *data realism*, the extent to which the synthetic datasets resemble and reflect patterns in real datasets, *utility*, measured by the performance on predictive tasks, and *privacy*, evaluated by the risk of identification of patients or attributes in the original data.^20^

### Non-LLM approaches and unresolved challenges

Standard synthetic data generation methods seek to simulate the generating process of the original data through an estimation of the original data distributions. Classical statistical approaches include kernel density estimation^33^ and Markov Chain Monte Carlo.^34^ However, these methods often impose limiting assumptions on the data distribution, which precludes estimation of the complex correlation structure typically found in medical data.^20^ Popular state-of-the-art methods include Generative Adversarial Networks (GANs)^35^ and variational autoencoders.^36^ GANs are composed of 2 neural networks, a “generator” and a “discriminator,” trained in tandem. The generator’s objective is to create synthetic data indistinguishable from the real data, and the discriminator’s objective is to differentiate between the generated and real data. Variational autoencoders are also composed of 2 neural network models: the “encoder” aims to compress an original dataset into a latent representation and the “decoder” aims to decompress the latent representation back to the original data. The decoder can then be used to generate synthetic data. These methods have been used to generate a variety of health data types, including both snapshot and longitudinal electronic health record (EHR) data, sociodemographic data, lab/measurement data, and image data.^30^^,^^37–41^ We provide illustrative applications of these methods with recent examples from the literature.

Li et al^42^ developed a GAN-based model to simultaneously generate multiple types of clinical time series data. After training on 141 488 unique patients’ intensive care unit (ICU) data, they were able to synthesize sequences of patients’ health indicators, including oxygen level, blood pressure, and heart rate. They demonstrate that augmenting their real training data with synthetic data to increase training data size improves performance on a downstream task of predicting patients’ need for mechanical ventilation or vasopressors. However, this is achieved through significant data pre-processing, including imputation, smoothing, and truncation of time series. Biswal et al^43^ proposed a variational autoencoder model to generate patients’ clinical encounters and their corresponding features, including diagnoses, medications, and procedures. The authors were able to generate longitudinal encounter sequences for a chosen disease. The paper highlighted that complex longitudinal clinical interactions can be generated, but with the limitation of not being able to account for comorbidities from more than 1 disease. Collectively, the literature suggests that previous approaches can generate a wide variety of health data types, can focus on generation for specific health conditions, and incorporate privacy into model training.^44^ A variety of other models have also been developed to handle varying time periods between clinical visits,^45^ missing or incomplete original data,^46^ and incorporation of disease-specific domain knowledge.^47^ However, these models each have their drawbacks, such as their limited focus on 1 data modality or on 1 disease, or their inflexible data pre-processing requirements.

However, many technical challenges remain. Hernandez et al^26^ and Ghosheh et al^31^ observe that current GAN-based approaches are tailored to specific data structures and contexts, making generalization and transfer between contexts difficult. Ghosheh et al^31^ also point out GAN’s lack of ability to generate complex multimodal data, which has been shown to improve predictive performance.^48^ Augmentation of real data with synthetic data has the potential to improve multimodality models’ performance, yet current methods cannot generate data across data types. Murtaza et al^20^ similarly point out that while existing methods have shown proficiency at synthesizing disease-specific longitudinal EHR data, “generating comprehensive longitudinal records with co-morbidities remains an open challenge.” Another issue is the difficulty of incorporating expert knowledge into generation methods, whether in the form of disease progression models or constraints on clinical knowledge violation.^20^ Yan et al^49^ found in their benchmarking study that all tested models made mistakes in assigning gender-specific disease codes to varying degrees. LLMs have the potential to overcome these current limitations.

### Application of LLMs for SHDG

Although several studies have explored the use of LLMs for SHDG, both in the context of text-based tasks, such as generation or augmentation of clinical language,^50^^,^^51^ and tabular EHR data tasks,^52–54^ developments in this area are still nascent, focusing largely on proof-of-concept rather than field applications. Table 1 summarizes key recent studies investigating SHDG with LLMs. One of these studies, Yuan et al, ^50^ addressed the issue of matching patients, based on their EHR data, to clinical trials, accounting for the trials’ inclusion and exclusion criteria. Existing models generally have had limited success due to terminology discordance across the datasets, which renders matching more challenging. Thus, Yuan et al^50^ used ChatGPT to augment inclusion and exclusion criteria descriptions for clinical trials in order to facilitate improved matching. Tang et al^51^ focused on the challenge of acquiring labeled data for 2 text classification tasks: recognition of biomedical vocabulary, or “entities,” and extraction of relationships between those entities. They used ChatGPT to generate a synthetic dataset for these 2 tasks, incorporating a combination of prompt engineering (prompt engineering is the process of optimizing prompts given to interactive LLMs, such as ChatGPT, to achieve a certain task without having to further train the LLM) and human-labeled seed examples into their workflow. Borisov et al^52^ investigated the ability of LLMs to generate tabular data. They converted rows of heterogeneous features (both categorical and numerical) into sentence-like textual representation, fine-tuned (fine-tuning refers to a strategy of adapting pre-trained models to specific tasks, by using a dataset of labeled examples to update either the whole model, or by adding and training a relatively small number of additional layers) GPT-2 to generate similar synthetic text data, and then converted the synthesized text back into tabular data. Borisov et al,^52^ however, considered just 1 tabular health dataset, and did not evaluate privacy preservation characteristics. Seedat et al^53^ examined the capabilities of GPT4 to generate tabular data out-of-the-box, using a 3-section prompt with data context, data examples, and generation instructions and post-generation filtering for data quality. They found that GPT4 generates high-utility data from few examples, holding promise for health applications with low data availability (eg, rare diseases). Finally, Kim et al^54^ focus on synthetic data generation with LLMs in settings of outcome class imbalance, common in health settings. Using out-of-the-box generation with GPT4, Llama 2 ^58^ and Mistral,^59^ they find that prompts specifically identifying and partitioning examples from each class produce synthetic data that boost model performance for a minority class. Collectively, these studies highlight the potential of LLMs for multimodal synthetic data generation and generation from few training examples.

**Table 1.** Summary of current research applying LLMs to synthetic data generation.

Modality	Downstream application	LLM(s) used	Reference(s)
Text	Clinical trial-patient matching	GPT3	Yuan et al^50^
	Biomedical term comprehension	GPT3	Tang et al^51^
	Radiology report generation	GPT4	Xie et al^55^
	Alzheimer’s detection from EHR notes	GPT4	Li et al^56^
	Clinical NLP tasks (general purpose)	GPT3.5	Xu et al^57^
Tabular	Binary classification (general purpose)	GPT2	Borisov et al^52^
		GPT3.5, GPT4	^a^Seedat et al^53^
		GPT3.5, LLaMa-2-7b, Mistral-7b	^a^Kim et al^54^

a Not currently peer reviewed.

Abbreviation: LLM, large language model.

## Open research directions

Given the limited research to date on LLMs for SHDG, many important questions remain around the potential opportunities and risks. Building on and extending systematic reviews of synthetic data generation in healthcare^20^^,^^23^^,^^32–36^ and benchmarking studies,^49^^,^^60–63^ we describe useful avenues for further research below.

### Evaluation metrics

To fully understand the value potential of LLMs, it is important to establish a portfolio of metrics to evaluate the quality of the generated data and to compare synthetic data generating LLMs to the current state of the art. There is already a lack of standardization of metrics in the broader field of health data generation^20^^,^^26^^,^^28^ and metrics for LLM performance on clinical prediction tasks.^64^ Dimensions that are critical to measure include the aforementioned data realism, utility, and privacy, token usage, computational cost (including power consumption), and diversity. Researchers should aim to present results on benchmark datasets and metrics, as well as assessments specific to a potential deployment context.

Some evaluation criteria have established metrics, such as the predictive performance of a downstream machine learning (ML) model after augmentation with synthetic data, which is both common^28^ and considers a likely setting for EHR data use. Computational cost metrics have recently become more prevalent^26^ and are particularly important in the LLM context, given the high costs of training and prompting, whether monetary or environmental.^65^ Efficiency metrics, such as generation time, algorithmic complexity, and computing power required, can help organizations make economic assessments of synthetic data generation alternatives. When evaluating privacy, metrics should focus on risks specific to healthcare data, such as re-identification, using measurements like the ability to predict whether a real data point belonged to the generator’s training set.^28^ One must also consider unique vulnerabilities of the LLMs. For example, ChatGPT is vulnerable to prompt injection, where users design prompts to reveal sensitive data that ChatGPT is intended not to expose.^66^ Thus, a new privacy metric could evaluate whether the generating LLMs reveal private medical information across a range of prompts. Furthermore, given the breadth of LLM training data, new categories of risks must be anticipated, including inadvertent infringement of intellectual property or generation of toxic or harmful language.^67^

Given existing research questioning the value of general-purpose models over tailored clinical models,^68^ a comprehensive comparison of LLM-based methods to tailored methods for synthetic data generation across a portfolio of relevant metrics is critical.

### LLM adoption

**Figure 2.**
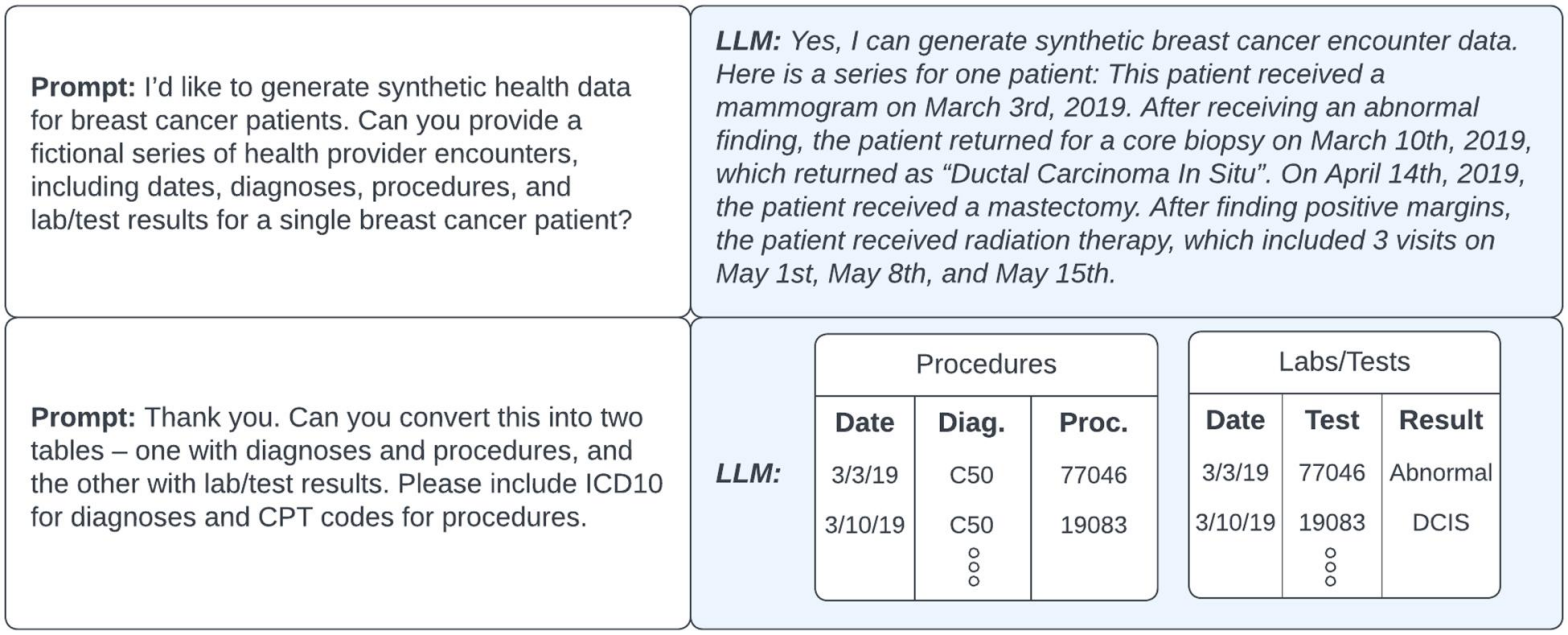
An example of synthetic health data generation with prompts to a large language model (LLM).

Choices related to the specific LLM model to be deployed as well as the generation approach are critical to understand. In the context of synthetic data, both prompt engineering and fine-tuning have already been applied.^51^^,^^52^ Prompt engineering can involve a variety of prompt templates and include either few or zero examples as part of the prompt^69^—an example of a zero-example prompt is shown in Figure 2. Similarly, one can explore or develop multiple different fine-tuning approaches.^70^ Furthermore, there are many existing LLMs, each of which may be more appropriate for certain approaches—fine-tuning may be easier with “smaller” LLMs such as Llama^71^ whereas prompt engineering is better suited for “larger” LLMs such as GPT-4. These choices can also be framed in the tradeoff between “buy” versus “build”—do the benefits of fine-tuning LLM models over direct application of out-of-the-box models outweigh the fine-tuning development cost? Extensive exploration of such strategic choices in adoption is necessary to make informed decisions for a specific context and task.

### Data efficiency

While the promise of synthetic data is clear, the practical feasibility of creating quality data must address the question of how much real data are necessary to generate high-fidelity synthetic observations. Given the large number of rare diseases and conditions (McDuff et al., 2023),^23^ well-documented difficulties of acquiring health data, the privacy risks involved with sharing increasing patient data with models, and the potential costs of fine-tuning LLMs with increasing data,^72^ there is great interest in maximizing the data efficiency of synthetic data generating LLMs. However, providing fewer examples can lead to decreased generating ability,^42^ particularly of rare real cases that are all the more important to have represented in synthetic data. Yet, LLMs have a crucial advantage in this environment. As shown by Seedat et al,^53^ LLMs can leverage prior knowledge from training to generate synthetic tabular data from very few examples. Application of their and related methods to health data holds great promise for the many low data contexts in healthcare, that should be explored.

### Generalization

As previously noted, non-LLM-based models for synthetic data generation struggle to generalize, whether in handling multiple data modalities, transferring between health contexts, or incorporating domain knowledge.^26^^,^^31^ Because LLMs are general purpose models trained on diverse knowledge bases, they are well-equipped to handle each of these challenges. Borisov et al^52^ showed that LLMs can simultaneously generate different data modalities with little tailoring, whether discrete, continuous, or categorical text data. Gruver et al^73^ demonstrated that LLMs can generate time series forecasts, while also handling missing data and creating textual explanations of predictions. Additionally, there are many existing LLMs trained specifically on medical text corpuses such as ClinicalBERT, Med-PaLM, and GatorTron which have the potential to automatically incorporate their domain knowledge into data generation.^74–76^ Thus, an important question for future work is a deeper understanding of if and how LLMs can convert knowledge from their training data into generalization across data modalities and contexts.

### Health equity

Another critical area that warrants further research is the risks and opportunities of synthetic data generation with LLMs in the domain of health equity. One clear risk is the perpetuation of existing biases in the data used to train LLMs and biases in health data^77^^,^^78^—Bhanot et al^79^ have documented violations of fairness metrics in existing synthetic data generation methods. For instance, they found that the realism of synthetic sleep data was not equal across age groups.

However, as noted by McDuff et al,^23^ synthetic data generation also provides opportunities to correct existing health equity issues. For instance, machine learning models often underperform for minority groups due to a lack of training data.^80^ Racial minorities are underrepresented in clinical trials and in health data^81^—synthetic data generation can help increase representation of smaller groups in augmented datasets and boost performance of these models for underrepresented groups. Thus, 1 valuable research direction is to compare the boost in performance for minority groups that LLM-based synthetic data provide over other common ML fairness methods aimed at improving performance for small groups.

### Regulatory challenges

Moving forward, the regulatory environment for generative AI will likely evolve rapidly. Regulation of LLMs in general, and synthetic health data in particular, is not clearly delineated in current data protection guidelines (eg, The General Data Protection Regulation [GDPR]).^82^ However, privacy preservation is a key policy requirement in emerging legislation on AI such as the EU AI Act^83^ and the Executive Order issued in the United States.^84^ Giuffrè et al^85^ discuss the difficulty of proving whether synthetic data are fully anonymized, as required by GDPR, as recent methods claiming to achieve de-identification were shown to retain vulnerability to re-identification attacks. One must also consider the privacy policies of LLM providers. For example, OpenAI’s policies around storing user data, training future models on user data, and sharing data with third parties may preclude researchers from providing sensitive patient data in input prompts.^86^ There are also considerations for the data used to validate the performance and safety of medical devices, which regulators must consider; relatedly, the Food and Drug Administration (FDA) and the Advanced Research Projects Agency for Health (ARPA-H) recently launched a program to help facilitate access to broader diversity in training and test data to better empower FDA pre-market submissions.^87^ The intersection between the regulatory landscape and the use of LLMs for SHDG represents a final opportunity for further work.

## Conclusion

LLMs have already shown great promise in a variety of healthcare applications, and SHDG is a logical next high-impact application area of LLMs. These methods can provide opportunities to address persistent challenges such as fairness in health modeling. However, we must ensure that potential drawbacks to LLM-based models are carefully examined as well. Each of these concerns—privacy, data efficiency, or bias perpetuation—is an important area of research as we develop new LLM-based synthetic health generation approaches.

## Data Availability

There are no new data associated with this article.
